# BEHAVIORAL AND PSYCHOLOGICAL SYMPTOMS OF DEMENTIA (BPSD) IN HOSPITALIZED OLDER ADULTS WITH AD/ADRD

**DOI:** 10.1093/geroni/igac059.1506

**Published:** 2022-12-20

**Authors:** Allison Marziliano, Alex Makhnevich, Edith Burns, Michael Diefenbach, Liron Sinvani

**Affiliations:** Northwell Health, Manhasset, New York, United States; Northwell Health, Manhasset, New York, United States; Northwell Health, Manhasset, New York, United States; Northwell Health, Manhasset, New York, United States; Northwell Health, Manhasset, New York, United States

## Abstract

The epidemiology of Behavioral and Psychological Symptoms of Dementia (BPSD) in hospitalized older adults with Alzheimer’s Disease (AD) and AD-related dementias (ADRD) has not been well-characterized. The purpose of this abstract is to examine the prevalence, patient-level factors and hospital outcomes associated with BPSD in hospitalized older adults with AD/ADRD. Data was extracted from the electronic health record (EHR) of older adults (aged 65+ years) with AD/ADRD, admitted to one of 7 hospitals in the greater New York metropolitan area during 2019. Three independent coders reviewed the EHR and recorded the presence or absence of the 11 domains of the Neuropsychiatric Inventory (NPI), a validated measure of BPSD. Of N = 1,865 hospitalized older adults with AD/ADRD, 1,564 had BPSD (prevalence = 83.9%). The most commonly reported BPSD were agitation (72.3%) and anxiety (17.7%). Older age (M = 84.6, SD = 7.6 versus M = 82.4, SD = 7.8, p = .000) and antipsychotic medication use prior to hospital admission (91% of the older adults who used home antipsychotics had BPSD, p = 000) was associated with BPSD. With regard to hospital outcomes, BPSD was associated with: increased mortality (of those who died, 90.6% had BPSD, p = .006), and increased likelihood of readmission to the hospital within 30 days of discharge (of those readmitted, 89.3% had BPSD, p = .007). Given its high prevalence and associated poor outcomes, recognizing and managing BPSD in hospitalized patients with AD/ADRD is critical to improving quality of care for this vulnerable population.

